# Supplementary material for: Aligning cellular and molecular components in age-dependent tertiary lymphoid tissues of kidney and liver
Source: PLoS One. 2025 Feb 27;20(2):e0311193. doi: 10.1371/journal.pone.0311193 (PMC11867392; doi:10.1371/journal.pone.0311193)
Supplement: S1 Table — (DOCX) [file pone.0311193.s001.docx]

**Supplemental Table 1: Primer sequences used for real-time PCR**

| Gene | Sequence (5′–3′) | |
| --- | --- | --- |
|  | Forward | Reverse |
| *Gapdh* | ACGGCAAATTCAACGGCACAGTCA | TGGGGGCATCGGCAGAAGG |
| *Cd3e* | TGGCTACTACGTCTGCTACACAC | ACCTCCACACAGTACTCACACAC |
| *Cd19* | CCTGGGCATCTTGCTAGTGATTG | AAGCATTCCACCGGAACATCTC |
| *Cxcl13* | CGTGCCAAATGGTTACAAAGATT | GTGGCTTCAGGCAGCTCTTC |
| *Ccl19* | CCTGGGAACATCGTGAAAGC | TGGAGGTGCACAGAGCTGATA |
| *Des* | AGCCAGGCCTACTCGTCCAGCCA | CCGCCCGACGTGCGCGACACCTG |
| *Ngfr* | GCAGAGCGGACTGAGCTAGAA | CTTGGCACCTCCAAAGGACA |
