# Supplementary figures and images for: Aligning cellular and molecular components in age-dependent tertiary lymphoid tissues of kidney and liver
Source: PLoS One. 2025 Feb 27;20(2):e0311193. doi: 10.1371/journal.pone.0311193 (PMC11867392; doi:10.1371/journal.pone.0311193)

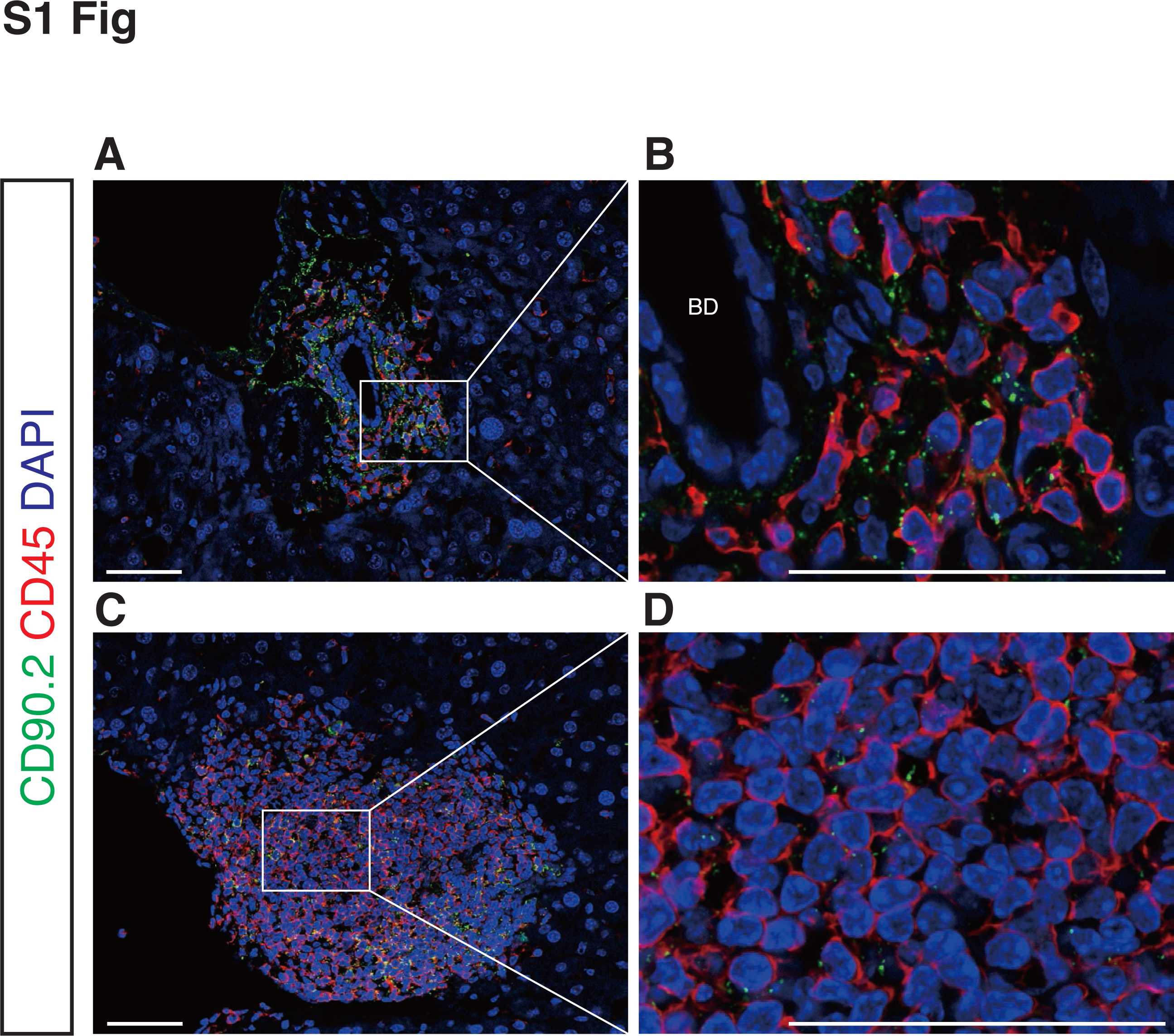

Supplement: S1 Fig — Immunofluorescence of CD90.2 (a portal fibroblast and a T-cell marker) and CD45 around bile ducts (A, B) and within TLTs (C, D). Abbreviation: BD, bile duct. Scale bars; 50 μm. (TIF) [file pone.0311193.s003.tif]

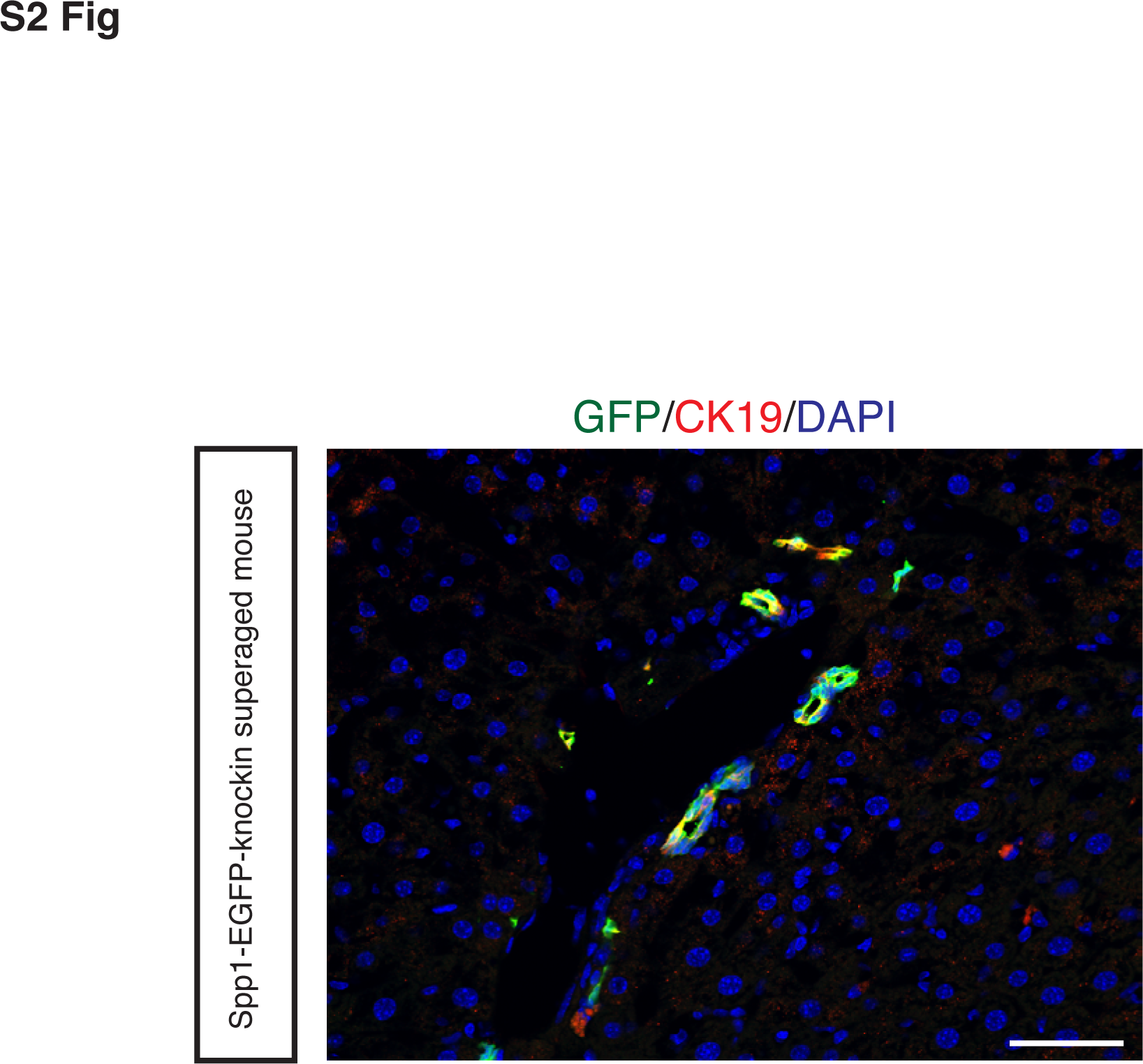

Supplement: S2 Fig — Immunofluorescence of GFP and cytokeratin (CK) 19 (a bile duct marker) in the liver of superaged Spp1-EGFP-Knockin mice. EGFP is visualized with an anti-GFP antibody. Scale bar: 50 μm. (TIF) [file pone.0311193.s004.tif]

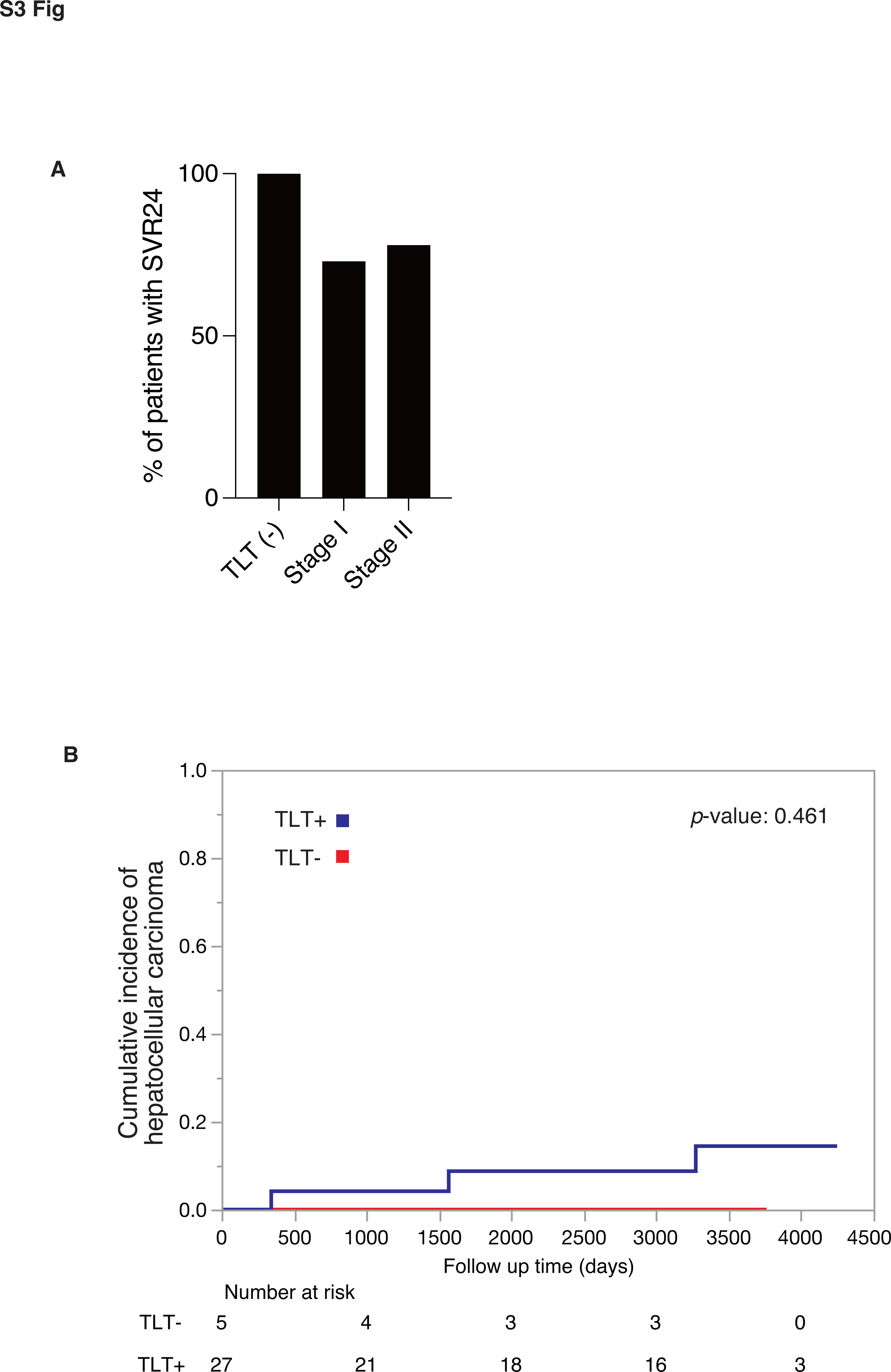

Supplement: S3 Fig — (A) The proportion of patients achieving sustained virological response at week 24 (SVR24) after interferon treatment in patients without TLTs, with Stage I TLTs and with Stage II TLTs (n = 5, 11 and 9, respectively). The proportion of patients with SVR24 in patients without TLTs, with Stage I TLTs and with Stage II TLTs are 100%, 73% and 78%, respectively. (B) Cumulative incidence of hepatocellular carcinoma (HCC) in patients with or without TLTs after liver biopsy (n = 27 and 5, respectively). The cumulative incidences of HCC in patients with TLTs are 4.17, 4.17, and 8.73% at 1, 3, and 5 years after the liver biopsy, respectively. Those without TLTs are 0, 0, and 0% at 1, 3, and 5 years after the liver biopsy, respectively. Log-rank test is used to compare the cumulative incidence of HCC. (TIF) [file pone.0311193.s005.tif]
