# Supplementary material for: Aligning cellular and molecular components in age-dependent tertiary lymphoid tissues of kidney and liver
Source: PLoS One. 2025 Feb 27;20(2):e0311193. doi: 10.1371/journal.pone.0311193 (PMC11867392; doi:10.1371/journal.pone.0311193)
Supplement: Attachment — Submitted filename: point_by_point 240824 FInal_NT.docx [file pone.0311193.s006.docx]

**Point by point reply to the Editor and reviewers’ comments and suggestions**

We would like to thank the editors and the reviewers for their valuable and insightful comments and the useful suggestions that have helped us to improve our manuscript.

*- Visualization of obtained results must be improved*

**Reply to the academic editor:**

Thank you for your important comments. We paid attention to the visualization quality of newly added data (Fig. 1R-S, S3_Fig)

*- Motivation behind proposed research should be more clearly explain. Please elaborate what is "beyond state-of-the-art" of proposed study.*

**Reply to the academic editor:** I appreciate your important comment. We have previously clarified the cellular and molecular components and clinical significance of TLTs in the kidney, and are curious whether our findings on renal TLTs could also be shared across the tissue. In this study, we therefore analyzed the cellular and molecular components of TLTs in the liver and their clinical significance, and found that there are similarities with TLTs in the kidney. We add the following sentence in the revised manuscript:

“Although TLTs develop with aging in other organs as well, their cellular and molecular components, and clinical significance remain unclear.” (p.2, l.7-8)

“we tried to characterize the cellular and molecular components of hepatic TLTs using superaged mouse and human livers, and to elucidate the clinical relevance of them.” (p.4, l.2-3)

“demonstrate that the cellular and molecular components of hepatic TLTs are similar to those of renal TLTs and” (p.20, l.18-19)

*- Make sure that the source code is available according to PLOS ONE publication policies.*

**Reply to the academic editor:** Thank you for your suggestion. We have no source code for sharing.

*- Make sure that you have conducted rigid statistical analysis.*

**Reply to the academic editor:** Thank you for your suggestion. We performed statistical analysis in Table 1. In Table 2, Fig4 L, N-0, we could not perform statistical analysis because of the existence of a zero cell count.

*- Please ensure that your manuscript meets PLOS ONE's style requirements, including those for file naming. The PLOS ONE style templates can be found at*
[*https://journals.plos.org/plosone/s/file?id=wjVg/PLOSOne_formatting_sample_main_body.pdf*](https://journals.plos.org/plosone/s/file?id=wjVg/PLOSOne_formatting_sample_main_body.pdf) *and*
[*https://journals.plos.org/plosone/s/file?id=ba62/PLOSOne_formatting_sample_title_authors_affiliations.pdf*](https://journals.plos.org/plosone/s/file?id=ba62/PLOSOne_formatting_sample_title_authors_affiliations.pdf)

**Reply to the academic editor:** Thank you for your suggestion. We followed the PLOS ONE’s style requirements.

*2. Please provide additional details regarding participant consent. In the ethics statement in the Methods and online submission information, please ensure that you have specified what type you obtained (for instance, written or verbal, and if verbal, how it was documented and witnessed). If your study included minors, state whether you obtained consent from parents or guardians. If the need for consent was waived by the ethics committee, please include this information.*

**Reply to the academic editor:** Thank you for your comment. We added the following sentence in the “Material and methods” section in the revised manuscript:

“Informed consent was obtained using an opt-out process on the website. Minors were not included among the patients.” (p.8, l.11-12)

*3. Thank you for stating the following financial disclosure: 
 [This research was supported by the Japan Agency for Medical Research and Development (AMED) under Grant Number AMED-CREST 19gm0610011, 22gm1210009, 22zf0127003h001, 22lm0203006, and 22ek0310020h0001; KAKENHI Grant-in-Aids for Scientific Research B (20H03697). This work was partly supported by the World Premier International Research Center Initiative (WPI), MEXT, Japan.].  
Please state what role the funders took in the study.  If the funders had no role, please state: "The funders had no role in study design, data collection and analysis, decision to publish, or preparation of the manuscript." 
If this statement is not correct you must amend it as needed. 
Please include this amended Role of Funder statement in your cover letter; we will change the online submission form on your behalf.*

**Reply to the academic editor:** Thank you for your suggestion. We added the following statement in the revised manuscript and cover letter.

"The funders had no role in study design, data collection and analysis, decision to publish, or preparation of the manuscript." (p.21, l.10-11)

*4. In the online submission form, you indicated that [The datasets of mice used and analyzed in the current study are available from the corresponding author upon reasonable request. The human datasets that support the findings of this study are available from the corresponding author, but restrictions apply to the availability of these data, which were used under license for the current study, and so are not publicly available. Data are however available from the authors upon reasonable request and with permission of the ethics committee at Niigata University and the ethical committee at Kyoto University Hospital. Previously published RNA-seq data are available in GEO (GSE84346:* [*https://www.ncbi.nlm.nih.gov/geo/query/acc.cgi?acc=GSE84346*](https://www.ncbi.nlm.nih.gov/geo/query/acc.cgi?acc=GSE84346) *and GSE171294:* [*https://www.ncbi.nlm.nih.gov/geo/query/acc.cgi?acc=GSE171294).*](https://www.ncbi.nlm.nih.gov/geo/query/acc.cgi?acc=GSE171294).)*]. 
All PLOS journals now require all data underlying the findings described in their manuscript to be freely available to other researchers, either 1. In a public repository, 2. Within the manuscript itself, or 3. Uploaded as supplementary information.
This policy applies to all data except where public deposition would breach compliance with the protocol approved by your research ethics board. If your data cannot be made publicly available for ethical or legal reasons (e.g., public availability would compromise patient privacy), please explain your reasons on resubmission and your exemption request will be escalated for approval.*

**Reply to the academic editor**: Thank you for your suggestion. We included the mouse datasets in the supplement. However, as for human dataset, the ethics committees at Niigata University and Kyoto University Hospital have not approved uploading the human datasets because public availability would compromise patient privacy. Still, data are available from the authors upon reasonable request and with permission of the ethics committees at Niigata University and Kyoto University Hospital.

We changed the data availability statement as follows:

“The mouse datasets are available in the Supporting Information files (S2_Table).” (p.22, l.9)

“The institutional contacts are: ethics@adm.niigata-u.ac.jp (the ethics committee at Niigata University) and ethcom@kuhp.kyoto-u.ac.jp (the ethical committee at Kyoto University Hospital).” (p.22, l.14-15)

*5. We note that Figures 1, 2, and 4 in your submission contain [map/satellite] images which may be copyrighted.*

**Reply to the academic editor**: We apologize for not being clear for copyright. All figures included in our paper were taken by the authors and are not copyrighted by another organization.

*Reviewer: 1
Comments to the Author
The authors have demonstrated that TLTs developed in the liver with aging, and that their cellular and molecular components were similar to those in the kidneys, such as senescence-associated T cells and age-associated B cells. Additionally, the authors have shown that the expression of TLT-related genes was elevated in the liver biopsy samples from HCV-infected patients compared with those without HCV infection. TLT formation was also associated with liver injury and fibrosis.
The present study suggests that TLT formation is observed across the tissues over aging. Although the causal relationship between TLT formation, chronic inflammation and aging remains unclear, further understanding of this cross-organ phenomenon may lead to solutions to many diseases and health problems.

HCV-related chronic hepatitis is now actively treated and curable disease. Is TLT formation associated with treatment response or liver function outcome?*

**Reply to the reviewer 1**: Thank you for your valuable and supportive comments and important questions. Unfortunately, this study did not collect the data on the usage of direct acting antivirals (DAAs). Combination therapy with DAA and interferon has been available in Japan since 2011. Among the patients analyzed, 37 patients received liver biopsy before 2011, and the remaining 10 patients received liver biopsy after 2011. Of these 10 patients, four patients on treated with interferon therapy, but the use of DAA is unknown. On the other hand, there are data about the treatment response to interferon therapy (newly added S3_Fig). The sustained virologic response at 24 weeks (SVR24) after interferon therapy was 100% for patients without TLTs, 73% for patients with stage I TLTs, and 78% for patients with stage II TLTs (S3_Fig A). While we could not perform Fisher’s exact test due to zero cell counts, there might be a tendency for TLTs in the liver to be associated with poor response to interferon therapy (S3_Fig A). On the other hand, TLT formation was not associated with the cumulative incidence of hepatocellular carcinoma (S3_Fig B).

We add the following sentences and S3_Fig in the revised manuscript.

“Kaplan-Meier method and log-rank test were used to depict and compare the cumulative incidence of hepatocellular carcinoma (HCC).” (p. 9, l.22-23)

“We also investigated the relationship between hepatic TLTs and outcomes of 47 HCV patients. Among them, 25 patients were treated with interferon, and the ratio of patients who achieved negative for serum HCV RNA at 24 weeks after the end of interferon therapy (SVR24) was 73% for patients with stage I TLTs and 78% for patients with stage II TLT, compared with 100% for patients without TLTs (S3_Fig A). Among 47 HCV patients, we were able to follow the prognosis of 32 patients. Within five years after liver biopsy, TLT formation was not associated with the cumulative incidence of hepatocellular carcinoma (S3_Fig B).” (p. 17, l.8-14)

*Is TLT formation associated with other liver dysfunction or disease such as non-C virus hepatitis and alcoholic/non-alcoholic/drug-induced/primary biliary cholangitis?*

**Reply to the reviewer 1**: Thank you for your comment. As you suggested, TLT formation is associated with other liver disease such as HBV hepatitis (Link A et al. Am J Pathol. 2011;178(4):1662-75) autoimmune hepatitis, and primary biliary cholangitis (Pikarsky E et al. Gastroenterology. 2016;151(5):780-3, van Buuren N et al. JHEP Rep. 2022;4(1):100388).

We added the following sentence in the revised manuscript:

“Lymphoid aggregate, some of which are TLTs, can be found in patients not only with HCV but also with hepatitis B virus infection, autoimmune hepatitis, and primary biliary cirrhosis [41-43].” (p. 20, l.2-3) *P18 LL389 “liver fibrosis x” ◊ “liver fibrosis”*

**Reply to the reviewer 1**: Thank you for your kind comment and we are sorry for the typo. We corrected the sentence.

*Reviewer #2:*

*Comments to the Author*

*The present manuscript is about tertiary lymphoid tissues (TLT) in the liver. The authors found aging and HCV were associated with TLT formation in the liver, and might contribute to liver dysfunction and fibrosis. The topic is interesting, but some critical concerns should be addressed.

1. Which type of non-hematopoietic cells express CXCL13 in TLT in the liver tissue?*

**Reply to the reviewer 2:** Thank you for your important comment. Immunostaining analysis showed the colocalization of CXCL13 and Desmin and CD21, respectively (Fig. 1R, S), which suggest that Desmin^+^ fibroblasts and CD21^+^ follicular dendritic cells (FDCs) express CXCL13. We added the following sentence.

“CXCL13 was expressed in Desmin^+^ fibroblasts and CD21^+^ FDCs (Fig 1R, S).” (p. 10, l.17-18)

*2. The interpretation of results and conclusion are too strong because statistical analyses have not been performed in Tables 1 and 2. Also, it seems to be difficult to describe TLT increases and develop to more advanced stages based on the data in Figure 4L-O. I understand that they are due to small sample size, but this point is critical because we should discuss based on data.*

**Reply to the reviewer 2:** Thank you for your important suggestion, and we apologize for the too-strong interpretation. We performed statistical analysis in Table 1. Unfortunately, in Table 2, Figure 4L, and Figure 4N-O, we could not perform Fisher’s exact test due to the existence of zero cell counts. Furthermore, we modified the result and discussion sections to avoid strong statement on the data without statistical significance.

We changed the result and discussion as follows.

“The median age of patients in each group was 53 years for patients without TLTs, 62 years for patients with stage I TLTs, and 63 years for patients with stage II TLTs, respectively (Table 1). While none of the patients without TLTs have been previously treated with interferon, some of the patients with TLTs have been previously treated with interferon. In addition, ALBI scores were −3.03 for patients without TLTs, −2.90 for patients with stage I TLTs and −2.73 for patients with stage II TLTs. Furthermore, FIB-4 index was 1.59 for patients without TLTs, 2.61 for patients with stage I TLTs and 2.55 for patients with stage II TLTs.” (p. 15, l.15-21)

“Overall, 100% of the aged patients (60 years of age or over) were positive for hepatic TLTs, while 72.7% of the young patients were positive for hepatic TLTs (Fig. 4L). The number of TLTs in the liver biopsy samples was not statistically different between two groups (Fig. 4M). The percentage of patients with stage II TLTs was 52% in aged patients and 27% in young patients (Fig. 4N).” (p. 16, l.12- p. 17 l.1)

“The percentage of patients with severe fibrosis (F3) was 26.8% in patients with hepatic TLTs and 0% in patients without hepatic TLTs (Fig. 4O). All patients had no difference in activity grade (Table 2).” (p. 17, l.5-7)

“With a clear definition of TLTs, we found that hepatic TLTs were observed more frequently than previously considered, especially in aged patients, in whom TLTs were present in 100% of patients.” (p. 19, l.15-17)

“Our findings demonstrate that the cellular and molecular components of hepatic TLTs are similar to those of renal TLTs and suggest that hepatic TLTs might be associated with aging, liver injury and liver fibrosis.” (p. 20, l.18-19)

Although the association between TLTs and liver fibrosis was not statistically supported in Figure 4, we reserved the sentence because of the association in Figure 3. To soften our statement, we changed "are" to "might be".
